# Two new species of pontogeneiid amphipods (Crustacea, Senticaudata, Calliopioidea) from Korean waters

**DOI:** 10.3897/zookeys.635.10604

**Published:** 2016-11-23

**Authors:** Tae Won Jung, Jong Guk Kim, Seong Myeong Yoon

**Affiliations:** 1 Marine Biodiversity Institute of Korea, Seocheon 33662, Korea; 2 Department of Marine Life Science, Chosun University, Gwangju 61452, Korea; 3 Department of Biology, Chosun University, Gwangju 61452, Korea

**Keywords:** Amphipoda, Eusiroides pilopalpus, Paramoera dentipleurae, Korea, new species, taxonomy

## Abstract

Two new pontogeneiid amphipods, *Eusiroides pilopalpus* **sp. n.** and *Paramoera dentipleurae* **sp. n.**, from Korean waters are described and illustrated. *Eusiroides pilopalpus* **sp. n.** can be distinguished from congeners by the following characters: mandibular palp article 3 has brush-like setation, maxilla 2 has an inner plate that is not enlarged, and gnathopods 1 and 2 ischium has a well-developed anterior lobe. *Paramoera dentipleurae* **sp. n.** can be discriminated from congeners by the following characters: head anterior cephalic lobe is sinusoid, pereopods 5–7 are homopodous and slender, pereopods 6 and 7 basis are proximally lobed but distally diminished on posterior margins, and epimeron 3 is prominently expanded posterodistally.

## Introduction

The family Pontogeneiidae Stebbing, 1906, one of the largest taxa of Calliopioidea Sars, 1895, consists of 31 genera readily distinguishable from its closely related families Cheirocratidae d'Udekem d'Acoz, 2010 and Hornelliidae d'Udekem d'Acoz, 2010 by the presence of calceoli on antennae 1 and 2 (absent in the former), and from the family Calliopiidae Sars, 1895 by a deeply to weakly cleft telson (notched, emarginated or entire in Calliopiidae) (Lowry and Myers 2013).

Members of the genus *Eusiroides* Stebbing, 1888 share some plesiomorphic characters with littoral marine pontogeneiids such as setose palmar margins of gnathopods, stout homopodous pereopods, and linear, apically setose rami of the uropods (Bousfield and Hendrycks 1995). To date, this genus accommodates 16 nominal species widely distributed in coastal marine regions of warm-temperate and tropical areas of the Atlantic Ocean, the Indian Ocean, and the Pacific Ocean (Haswell 1879; Stebbing 1888; Giles 1890; Chevreux 1900; Walker 1904, 1909; Barnard KH 1932; Ledoyer 1978; 1982; McKinney et al. 1980; Hirayama 1985; Bellan-Santini and Ledoyer 1986; Bousfield and Hendrycks 1995). Near Korean waters, the following two *Eusiroides* species have been recorded: *Eusiroides japonica* Hirayama, 1985 originally described from Japan and *Eusiroides diplonyx* Walker, 1909 originally described from the Seychelles in the Indian Ocean and subsequently reported from China (Walker 1909; Hirayama 1985; Ren 2012).

The genus *Paramoera* was established by Miers (1875) as monotypic based on *Paramoera australis*. It is one of the largest groups of Pontogeneiidae including more than 50 species (Miers 1875; Staude 1995; Sidorov 2010). *Paramoera* members are mostly confined to marine and brackish water habitats; however, some of them live in fresh water or subterranean environments from temperate latitudes around the Pacific Ocean (Staude 1995; Kuribayashi and Kyono 1995; Sidorov 2010). In the Far East region, seven species of *Paramoera* have been recorded from marine habitats: *Paramoera udehe* (Derzhavin, 1930), *Paramoera koreana* Stephensen, 1944, *Paramoera brevirostrata* (Bulycheva, 1952), *Paramoera mokyevskii* (Gurjanova, 1952), *Paramoera tridentata* Bulycheva, 1952, *Paramoera hanamurai* Hirayama, 1990, and *Paramoera koysama* Kuribayashi & Kyono, 1995 (Stephensen 1944; Bulycheva 1952; Gurjanova 1952; Hirayama 1990; Kuribayashi and Kyono 1995; Labay 2012).

In Korean waters, three pontogeniids, *Eusiroides japonica*, *Paramoera koreana*, and *Pontogeneia rostrata* Gurjanova, 1938 have been recorded from marine habitats (Kim and Kim 1987, 1991). Here, further two new species are reported, *Eusiroides pilopalpus* sp. n. and *Paramoera dentipleurae* sp. n., from Korean waters with detailed descriptions and illustrations.

## Materials and methods

Samples were collected from the benthic zones using a sledge net (mesh size 300 µm, mouth size 120 × 45 cm). Specimens were initially fixed with 5% formaldehyde-seawater solution. They were preserved with 85% ethyl alcohol after sorting in the laboratory. Samples were stained with lignin pink dyes. Specimen appendages were dissected in a Petri dish filled with glycerol using dissection forceps and a needle under a stereomicroscope (SZH10; Olympus, Tokyo, Japan). They were mounted onto temporary slides using glycerol-ethanol solution or permanent slides using polyvinyl lactophenol solution. Drawings were performed under a light microscope (LABOPHOT-2; Nikon, Tokyo) with aid of a drawing tube. Definition of the term for ‘seta’ and its types follows those of Watling (1989). Type material was deposited in the National Institute of Biological Resources (NIBR), Incheon, Korea.

## Systematic accounts

### Order Amphipoda Latreille, 1816 Suborder Senticaudata Lowry & Myers, 2013 Superfamily Calliopioidea Sars, 1895 Family Pontogeneiidae Stebbing, 1906

#### Genus *Eusiroides* Stebbing, 1888

##### Eusiroides pilopalpus sp. n.

Taxon classificationAnimaliaAmphipodaPontogeneiidae

http://zoobank.org/5B037355-73B0-4F5A-94A3-CD907324FAD9

**Figure 1.**
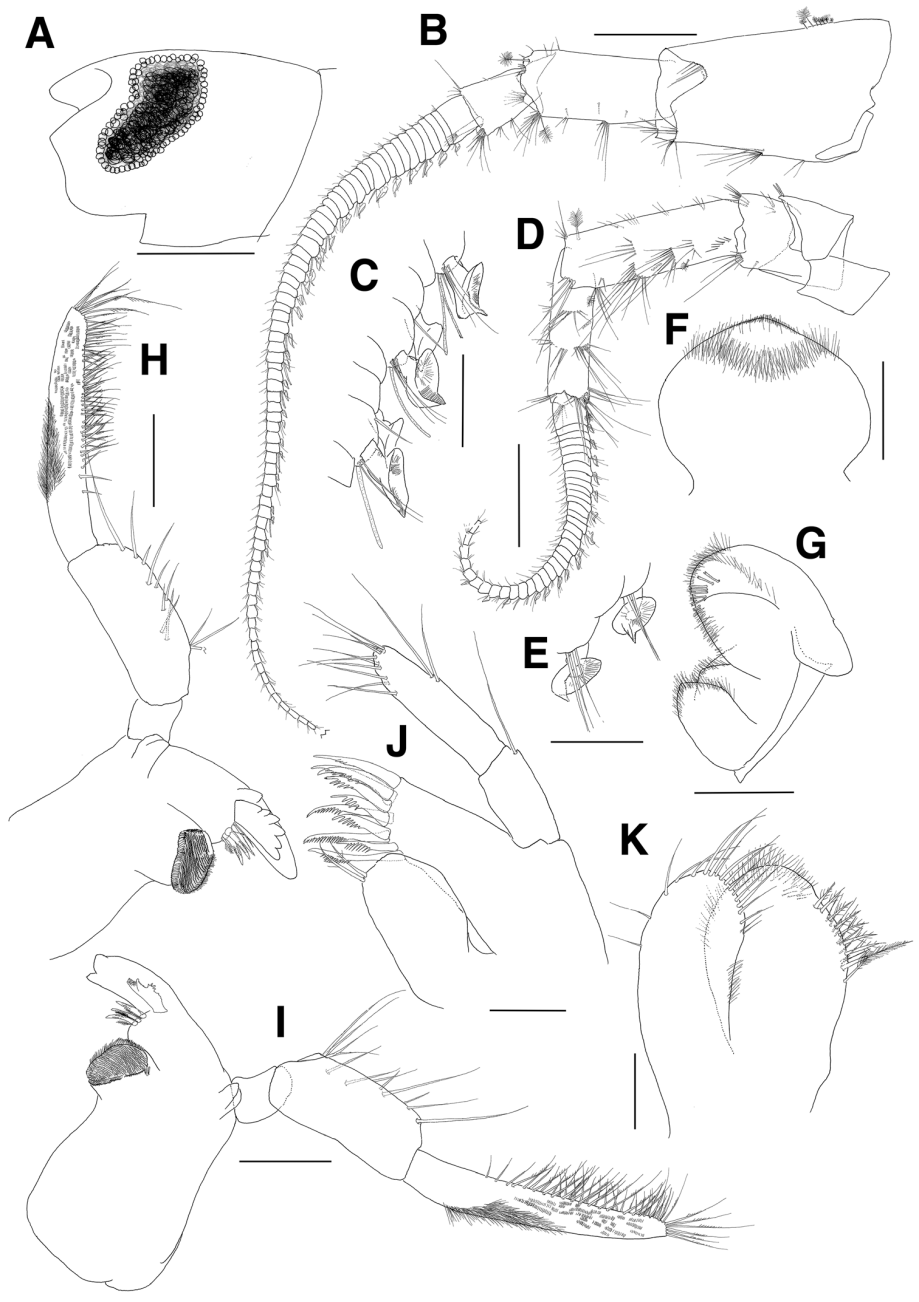
*Eusiroides pilopalpus* sp. n., holotype. **A** Head **B** Antenna 1 **C** Calceoli of flagellum on antenna 1 **D** Antenna 2 **E** Calceoli of flagellum on antenna 2 **F** Upper lip **G** Lower lip **H** Left mandible **I** Right mandible **J** Maxilla 1 **K** Maxilla 2. Scale bars 0.1 mm (**C, E, J, K**), 0.2 mm (**F–I**), 0.5 mm (**A, B, D**).

**Figure 2.**
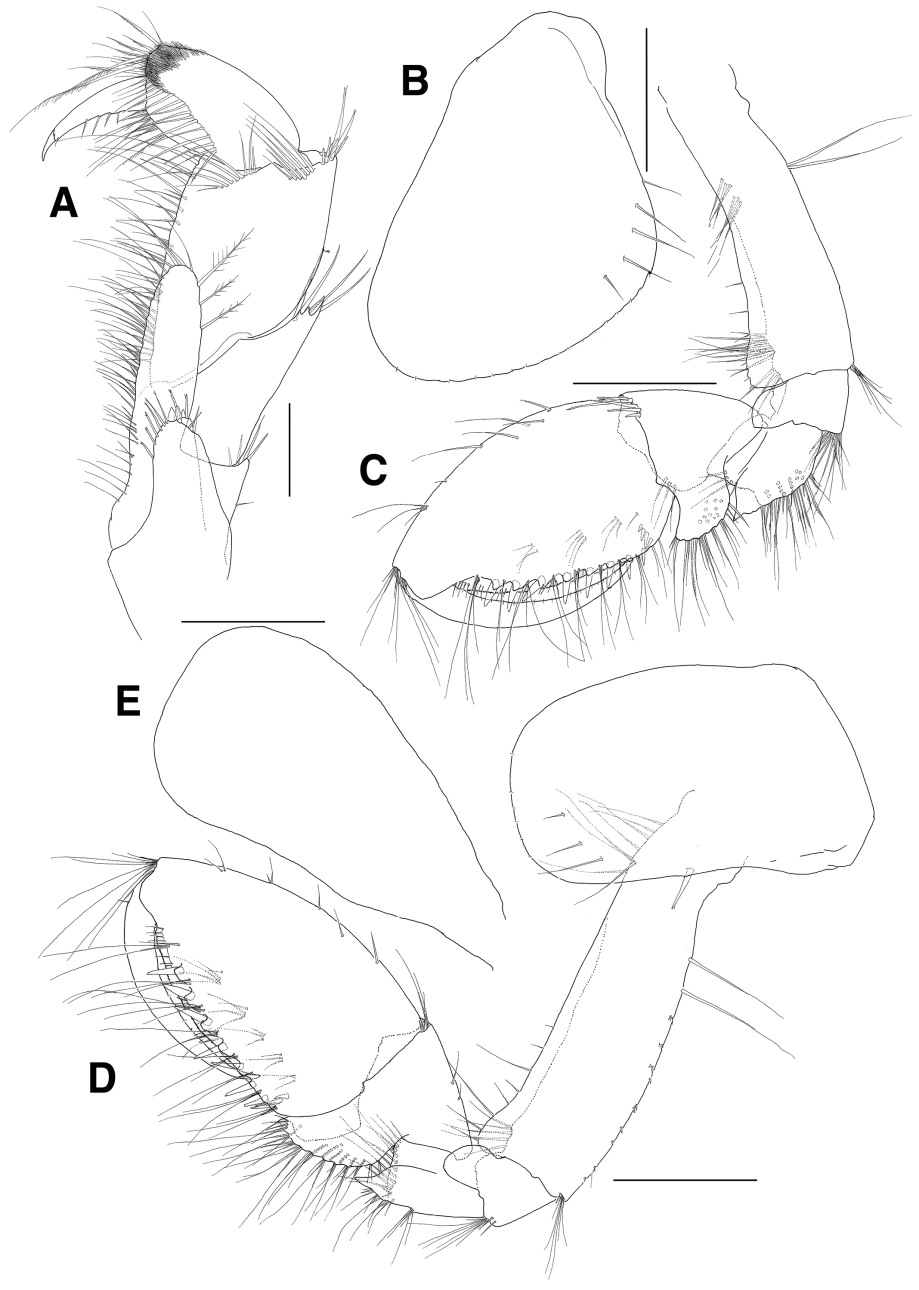
*Eusiroides pilopalpus* sp. n., holotype. **A** Maxilliped **B** Coxa 1 **C** Gnathopod 1 **D** Gnathopod 2 **E** Coxal gill of gnathopod 2. Scale bars 0.2 mm (**A**), 0.5 mm (**B–D**).

**Figure 3.**
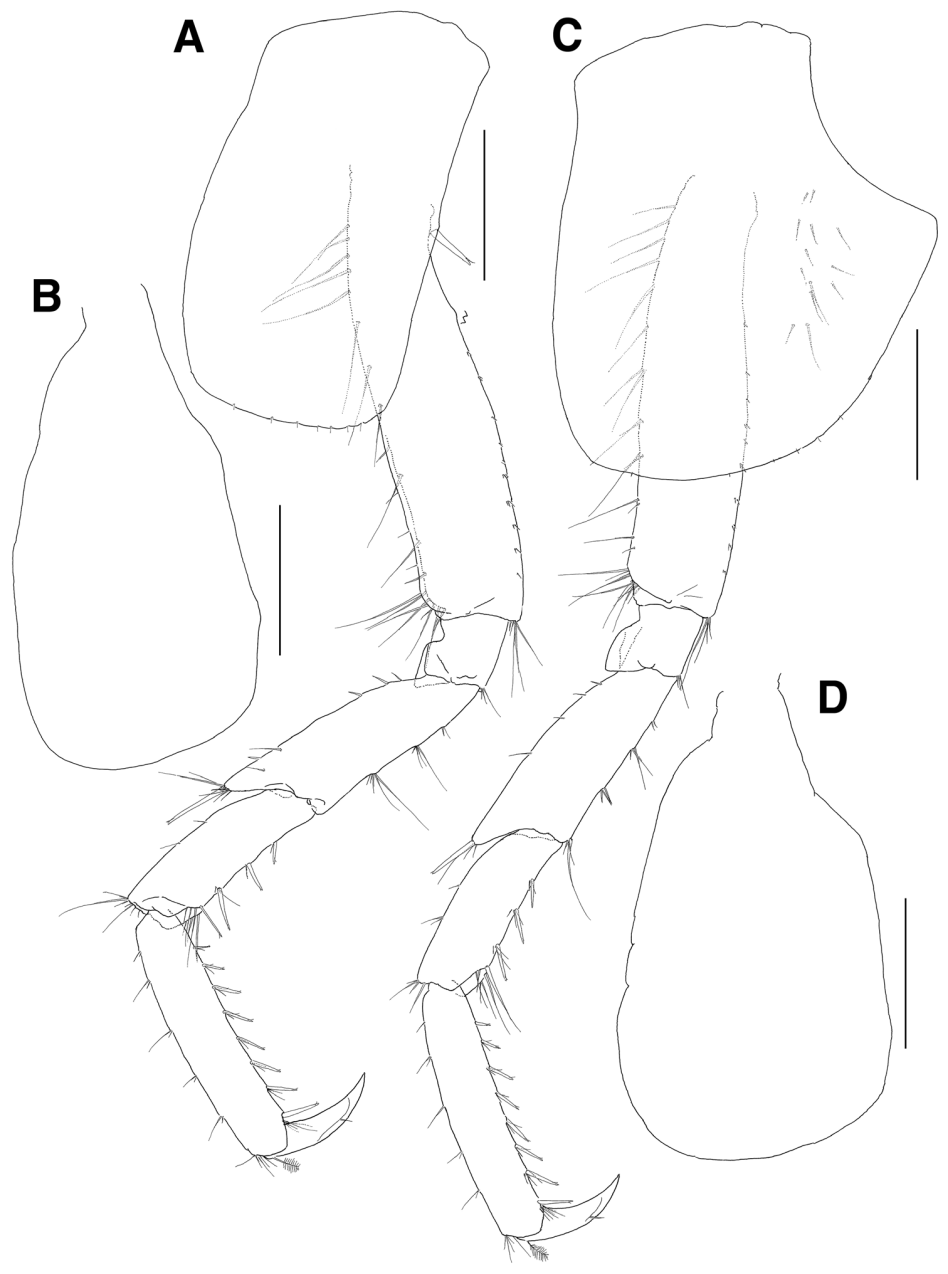
*Eusiroides pilopalpus* sp. n., holotype. **A** Pereopod 3 **B** Coxal gill on pereopod 3 **C** Pereopod 4 **D** Coxal gill of pereopod 4. Scale bars 0.5 mm.

**Figure 4.**
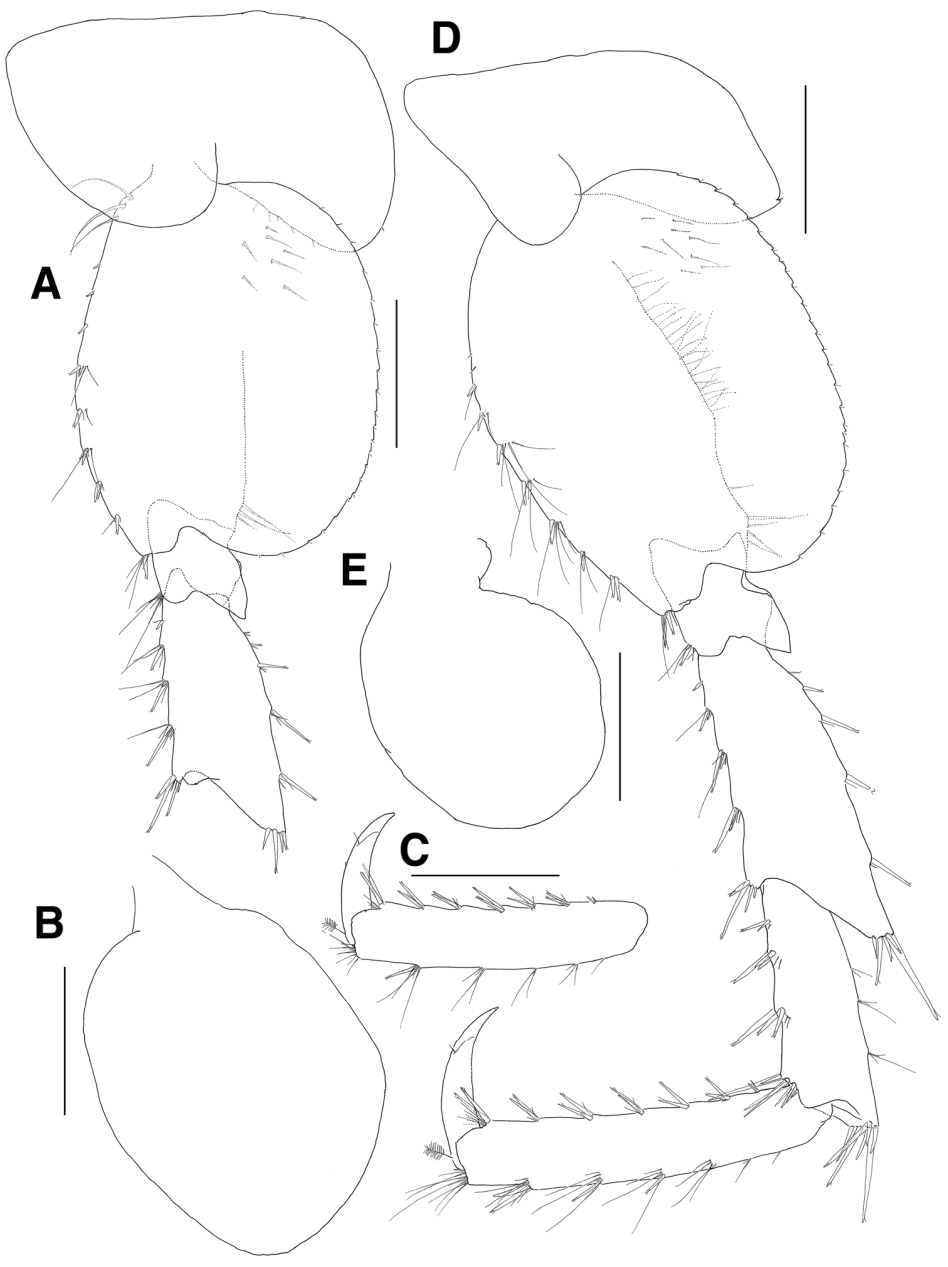
*Eusiroides pilopalpus* sp. n., holotype. **A** Pereopod 5 **B** Coxal gill of pereopod 5 **C** Propodus and dactylus on pereopod 5 **D** Pereopod 6. Scale bars 0.5 mm.

**Figure 5.**
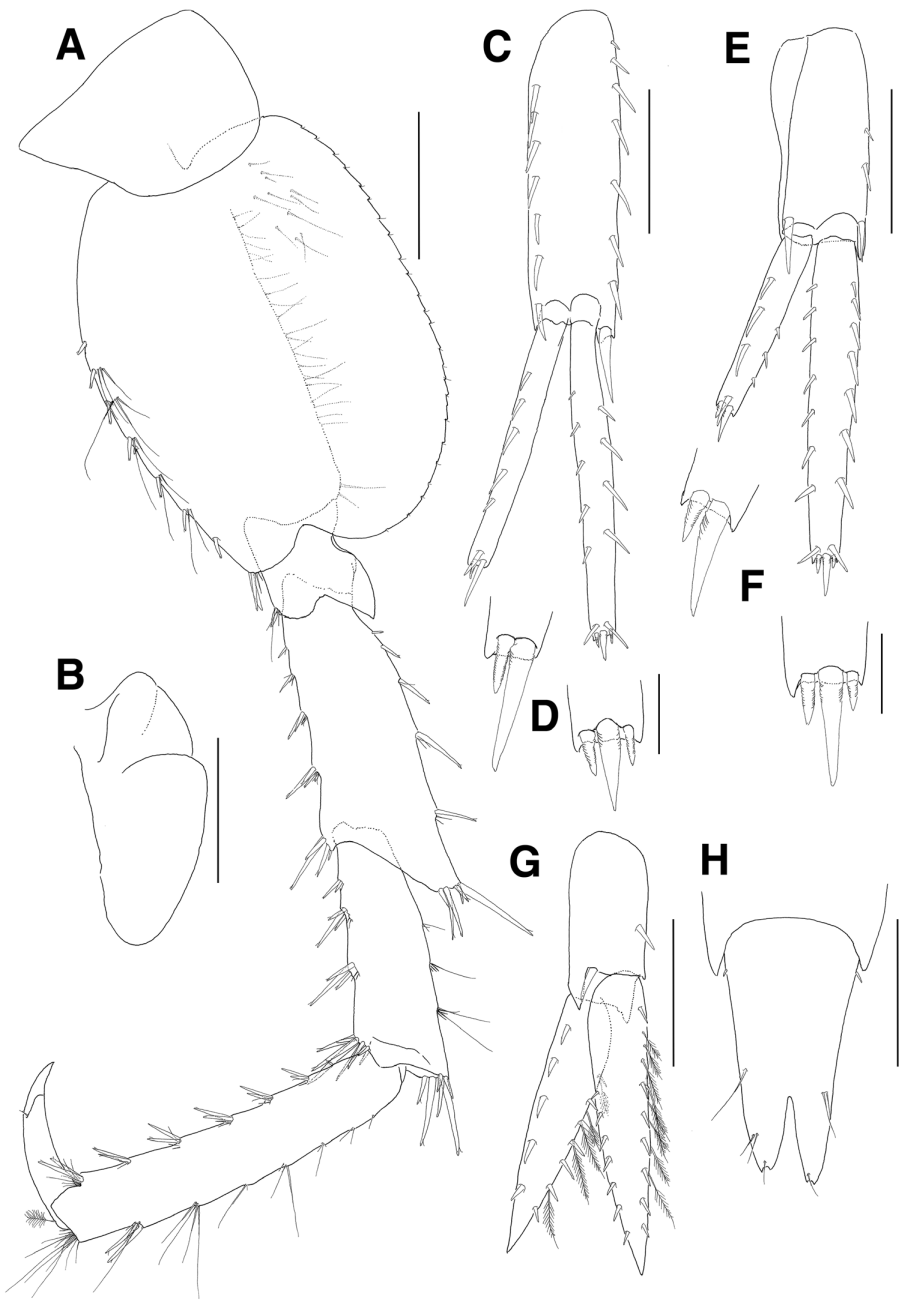
*Eusiroides pilopalpus* sp. n., holotype. **A** Pereopod 7 **B** Coxal gill of pereopod 7 **C** Uropod 1 **D** Apices of rami on uropod 1 **E** Uropod 2 **F** Apices of rami on uropod 2 **G** Uropod 3 **H** Telson. Scale bars 0.1 mm (**D, F**), 0.5 mm (**A–C, E, G, H**).

**Figure 6.**
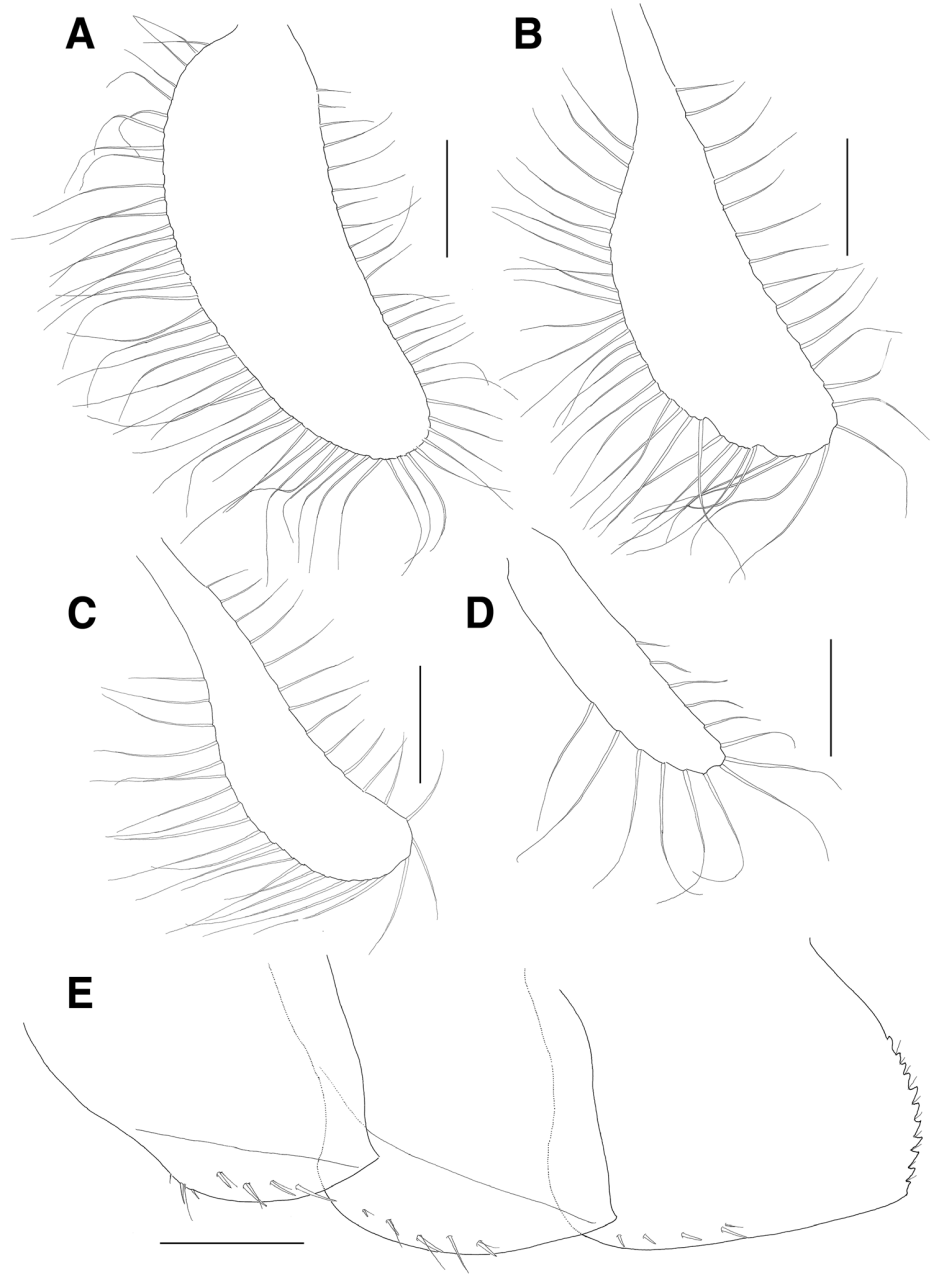
*Eusiroides pilopalpus* sp. n., holotype. **A–D** Oostegites of gnathopod 2–pereopod 5 **E** Pleonal epimera. Scale bars 0.5 mm.

Figs 1 , 2 , 3 , 4 , 5 , 6

###### Type locality.

Jeju Island, South Korea, 33°14'23"N 126°34'59"E, sublittoral (average depth 24 m).

###### Material examined.

Holotype: NIBRIV0000328601, adult female, 8.4 mm, collected from the type locality on 30 Nov 2012 by Prof. H.-Y. Soh.

###### Etymology.

The composite epithet of the specific name of *pilopalpus* is a combination of Latin *pilosus* and *palpus* meaning ‘hairy palp’. This name refers to the character of the mandibular palp article 3: the surface is covered with several rows of minute setae along the distal two-thirds length and with a group of brush-like fine setae at the center of the outer margin. Noun in apposition.

###### Diagnosis.

Head with short rostrum; eyes reniform, well-developed. Antennae with stout peduncular articles, with calceoli on flagellum; accessory flagellum of antenna 1 uni-articulate, as long as 1^st^ proximal article of flagellum. Upper lip slightly angulate distally. Lower lip outer lobe with 7 bifid setae on surface. Mandibles both with bi-dentate (1 small and 1 enlarged) incisors, with trifid and 6-dentate lacinia mobilis on right and left mandibles, respectively; palp article 3 covered with numerous fine setae laterodistally. Maxilla 1 outer plate with 10 dentate robust setae apically. Maxilla 2 inner plate broader than outer plate. Maxilliped with short inner plate; outer plate elongate; palp articles 1–3 expanded, article 4 falcate. Gnathopods moderately subchelate, similar to each other, with developed anterior lobe on basis and ischium; dactylus falcate, elongate. Pereopods 3–4 ordinary; basis lined with short setae posteriorly; ischium anterior lobe acute distally; merus anterodistal corner produced bearing 1 robust seta. Pereopods 5–7 basis expanded posteriorly; ischium posterior lobe developed; merus produced posterodistally. Pleonal epimera each with submarginal setae ventrally; epimera 2 and 3 with oblique redge on surface; epimeron 3 with serrations posteriorly. Uropod 1 peduncle with largest seta mediodistally; outer ramus with lateral setae only. Uropod 2 shorter than uropod 1, peduncle with 1 distal seta laterally. Uropod 3 rami lancerolate. Telson shallowly cleft (approximately 1/4 on its length).

###### Description of holotype female.

***Head*** (Fig. 1A): rostrum short; lateral cephalic lobes weakly produced and slightly oblique; antennal sinus not deep; eyes reniform, well-developed, enlarged.

***Antenna 1*** (Fig. 1B, C): stout, with length ratio of 1.0:0.7:0.3 in peduncular articles 1–3; peduncular article 1 with 1 group of 7 plumose setae on anterior margin proximally and with smooth groove bearing setae on lateral margin subdistally; peduncular article 2 with 1 subdistal and 2 distal setae laterally and 1 pair of distal setae medially on anterior margin, also with small groove bearing setae on distal margin; accessory flagellum uni-articulate, as long as 1^st^ proximal article of flagellum, with 5 setae on apex; flagellum more than 72-articulate and slightly longer than twice as long as peduncular articles 1–3 combined, proximal article longest, with single or paired aesthetascs and calceoli present from 1^st^ to 54^th^ articles discontinuously.

***Antenna 2*** (Fig. 1D, E): stout, shorter than antenna 1; with length ratio of 1.0:0.7 in peduncular articles 4–5; flagellum more than 36-articulate, slightly shorter than peduncular articles 3–5 combined, proximal article longest, with calceoli present from 1^st^ to 24^th^ articles discontinuously.

***Upper lip*** (Fig. 1F): apex convex, slightly angulate distally, covered with apical and subapical fine setae.

***Lower lip*** (Fig. 1G): inner lobe weak; outer lobe apically round with 7 bifid setae on surface; mandibular processes developed bearing round apices.

***Right mandible*** (Fig. 1I): incisor bi-dentate (1 small and 1 enlarged); lacinia mobilis trifid, each multidentate; accessory setal row with 3 serrate and 3 plumose setae alternatively; molar triturative, columnar; palp 3-articulate; palp article 1 shortest; palp article 2 rectilinear, with 12 setae on expanded medial margin; palp article 3 weakly falcate, with marginal and submarginal serrate setae on distal 3/4 of medial margin, covered with several rows of fine setae on distal 2/3 of surface, densely covered with group of brush-like setae at centre on lateral margin and surface, apex bluntly truncate with 6 serrate setae.

***Left mandible*** (Fig. 1H): incisor bi-dentate (1 small and 1 enlarged); lacinia mobilis 6-dentate; accessory setal row with 1 simple, 3 serrate, and 1 plumose setae; palp similar to that of right mandible.

***Maxilla 1*** (Fig. 1J): inner plate enlarged, with 2 plumose setae at mediodistal corner; outer plate with 10 dentate robust setae apically; palp long, beyond apical setae of outer plate, palp article 1 with 1 long seta laterodistally, article 2 with 1 row of 9 setae along apical and mediodistal margins, with 1 pair of long setae laterally.

***Maxilla 2*** (Fig. 1K): inner plate ovoid, broader than outer plate, with 1 row of 8 weakly plumose and 1 long plumose setae on medial margin subdistally, covered with fine setae on apical and subapical margins; outer plate with 16 setae on apical margin, with 2 setae on lateral margin distally.

***Maxilliped*** (Fig. 2A): inner plate short, tongue-shaped, with 3 robust setae apically, with 8 bifid and 2 simple setae arranged from apex to mediodistal margin; outer plate elongate, 2.0 times as long as inner plate, lined with numerous long setae arranged from apex to medial margin, with 3 long weakly plumose setae on lateral margin; palp with expanded articles 1–3; article 1 with 1 group of 4 elongate setae laterodistally; article 2 lined with numerous simple long setae on medial margin, with 2 rows of setae subdistally, with 1 group of 6 unequal setae at laterodistal corner; article 3 0.3 times as long as article 2, with ledge bearing 1 row of long setae on surface, with 7 serrate and 4 simple setae at laterodistal corner, covered with numerous fine setae laterodistally; article 4 falcate, slightly shorter than article 3, with 4 setae along inner margin.

***Gnathopod 1*** (Fig. 2B, C): moderately subchelate; coxa expanded anterodistally, with 16 minute setae on round ventral margin, with 1 small notch bearing 1 minute seta posteroventrally, with 1 oblique row of 5 long setae on surface posteroventrally; basis lined with short setae and shallowly lobed anterodistally, with 2 groups of 3 submarginal setae proximally and with 2 groups of 9 subdistal and 3 distal setae medially on anterior margin, with 1 pair of elongate setae on posterior margin proximally and with 1 group of 6 setae at posterodistal corner; ischium largely lobed anteriorly, without setae on posterior margin and with 1 group of 11 setae posterodistally; merus forming groove anterodistally, with 1 row of 4 setae medially, with acute protrusion posterodistally, lined with numerous setae on posterior margin; carpus longer than merus, without setae on anterior margin and with 1 row of 5 setae at anterodistal corner, posterior margin broadly lobed laterally, weakly crenulate and with groups of serrate and simple setae and with 3 setae mediodistally; propodus ovoid, shorter than basis, with 1 group of 3 setae and 6 single setae on anterior margin and with 1 group of 9 setae at anterodistal corner, with 1 vertical row of 4 robust defining setae on medial surface near posterior margin, palm approximately 3.0 times as long as posterior margin and minutely pectinate, with 7 robust setae along toothed submargin; dactylus falcate, stout, long, fitting palm.

***Gnathopod 2*** (Fig. 2E, D): moderately subchelate, similar to gnathopod 1; coxa rectangular, not expanded anterodistally, with 1 oblique row of 4 elongate setae on surface posteroventrally, with 1 robust seta on posterior margin, ventral margin subrounded with 7 minute setae, coxal gill longer than coxa and expanded distally; basis shallowly lobed and lined with 8 setae anterodistally, with 1 group of 5 setae mediodistally on anterior margin, with 2 elongate setae proximally and lined with single or paired minute setae on posterior margin, with 1 group of 7 setae at posterodistal corner; ischium largely lobed and slightly dilated anterodistally, without setae on posterior margin and with 1 pair of subdistal and 1 group of 7 distal setae at posterodistal corner; merus forming groove anterodistally, with 1 row of 13 setae medially, with acute protrusion posterodistally; carpus longer than merus, with 2 setae on anterior margin and 1 group of 4 setae at anterodistal corner, posterior margin broadly lobed, weakly crenulate and with groups of simple setae; propodus triangular, shorter than basis, with 3 single and 2 pairs of setae on anterior margin and with 1 group of 8 setae at anterodistal corner, with 1 vertical row of 3 robust defining setae on medial surface near posterior margin, palm poorly defined, about 3.0 times as long as posterior margin and minutely pectinate, with 7 robust setae along toothed submargin; dactylus falcate, stout, long, fitting palm.

***Pereopod 3*** (Fig. 3A, B): coxa rectangular, similar to that of gnathopod 2, facial setae absent, with 7 minute setae on ventral margin, with 1 robust seta on posterior margin, coxal gill as long as coxa and expanded distally; basis slightly curved proximally, anterior margin lined with setae, anterodistal lobe weak with several setae, with more than 10 groups of minute setae on posterior margin; ischium short, anterodistally lobed, with 1 group of 3 setae at posterodistal corner; merus 0.7 times as long as basis, expanded anterodistally, with 3 proximal and 2 distal setae on anterior margin, anterodistal corner produced with 1 group of 1 robust and 6 simple setae, with 3 groups of simple setae on posterior margin; carpus not expanded, 0.9 times as long as merus, with 2 setae on anterior margin and with 7 setae on anterodistal corner, with 3 pairs of 1 robust and 1 simple setae on posterior margin, posterodistal corner oblique with 4 lateral and 3 medial setae; propodus 1.1 times as long as carpus, with 5 pairs of setae on anterior margin and with 1 group of 5 setae at anterodistal corner, with 7 groups of robust and simple setae on posterior margin and with 1 group of 1 locking seta and 4 simple setae at posterodistal corner; dactylus falcate, as long as half of propodus.

***Pereopod 4*** (Fig. 3C, D): coxa acutely produced backwards posteriorly; other articles similar to those of pereopod 3.

***Pereopod 5*** (Fig. 4A–C): coxa bilobed, posterior lobe narrower and more ventrally produced than anterior lobe, with 5 minute setae on ventral margin, coxal gill subovoid, smaller than that of pereopod 4; basis ovoid, with 3 elongate setae proximally and lined with 3 single setae and 5 groups of setae on anterior margin, anterodistal corner bluntly lobed with 1 group of 5 setae, posterior margin expanded and with 22 weak serrations bearing 1 minute seta, with 3 setae mediodistally, with 7 setae on medial surface proximally; ischium short, anterodistal corner weakly lobed downwards with 1 group of 6 setae, posterior margin largely expanded and slightly lurched posterodistally on lateral border; merus shorter than basis, with 4 groups of robust and simple setae on anterior margin and with 1 group of 5 robust setae at anterodistal corner, posterior margin expanded with 5 groups of robust and simple setae, posterodistal corner produced with 4 robust setae; propodus 1.4 times as long as merus, with 6 groups of paired setae on anterior margin, with paired locking setae and 3 simple setae at anterodistal corner, posterior margin irregularly setose with robust and simple setae, with 6 short setae at posterodistal corner; dactylus falcate, 0.3 times as long as propodus.

***Pereopod 6*** (Fig. 4D, E): coxa bilobed, smaller than that of pereopod 5, anterior lobe small, posterior lobe expanded backwards and with 1 notch bearing 1 minute seta posteroventrally, coxal gill circular and slightly smaller than that of pereopod 5; basis ovoid, larger than that of pereopod 5, with 2 minute setae proximally and 7 groups of robust and simple setae on anterior margin, anterodistal corner bluntly lobed with 1 group of paired robust setae and 3 simple setae, posterior margin expanded with 25 weak serrations bearing 1 minute seta, with 23 setae proximally and 5 setae distally on medial border, with 8 setae on medial surface proximally; ischium short, anterodistal corner weakly lobed downwards with 1 group of 1 robust and 5 simple setae, posterior margin largely expanded and slightly lurched posterodistally on lateral border; merus shorter than basis, with 1 robust seta and 3 groups of robust and simple setae on anterior margin, with 1 group of 4 robust setae at anterodistal corner, posterior margin expanded with 2 setae and 3 pairs of robust and simple setae, posterodistal corner produced with 5 robust setae; carpus not linear, slightly shorter than merus, with 3 groups of robust and simple setae on anterior margin and with 1 group of 8 robust setae at anterodistal corner, posterior margin broadly expanded with 3 groups of simple setae, posterodistal corner weakly produced with 1 group of 7 robust setae; propodus 1.3 times as long as merus, with 6 groups of paired robust and simple setae on anterior margin, with paired locking setae and 7 simple setae at anterodistal corner, posterior margin setose irregularly with robust and simple setae, with 8 setae at posterodistal corner; dactylus falcate, 0.5 times as long as propodus.

***Pereopod 7*** (Fig. 5A, B): as long as pereopod 6; coxa unilobed, ventrally convex, coxal gill present, longish subovoid, smaller than that of pereopod 6; other articles similar to those of pereopod 6 except for flattened posterior margin of basis.

***Pleonal epimera*** (Fig. 6E): epimeron 1 with 1 oblique ledge on surface, with 1 group of 2 robust and 2 simple setae on ventral margin anteriorly, with 3 pairs of robust and simple setae and 1 robust seta submarginally, weakly produced at posteroventral corner; epimeron 2 larger than epimeron 1, also with 1 oblique ledge on surface, convex ventrally and with 4 pairs of robust and simple setae and 1 robust seta submarginally on anterior half of ventral margin, weakly produced at posteroventral corner; epimeron 3 largest, flattened ventrally, with 3 single setae and 1 pair of robust and simple setae on anterior half of ventral margin, posterior margin expanded backwards and with 11 serrations bearing 1 minute seta on distal half.

***Uropod 1*** (Fig. 5C, D): peduncle with 7 marginal and 1 distal robust setae dorsomedially and 7 marginal robust setae dorsolaterally; inner ramus linear, as long as peduncle, with 4 medial and 5 lateral robust setae dorsally, apex blunt with 1 pair of robust setae subapically and 3 robust setae (1 large and 2 small) apically; outer ramus shorter than inner ramus, with 5 lateral robust setae dorsally; apex blunt with 1 robust seta subapically and 2 robust setae (1 large and 1 small) apically.

***Uropod 2*** (Fig. 5E, F): peduncle 0.7 times as long as that of uropod 1, with 2 marginal and 1 subdistal robust setae dorsomedially, with 1 distal robust seta dorsolaterally; inner ramus 1.6 times as long as peduncle, with 7 medial and 7 lateral robust setae dorsally; apex blunt with 1 pair of robust setae subapically and 3 robust setae (1 large and 2 small) apically; outer ramus much shorter than inner ramus, with 3 medial and 3 lateral robust setae dorsally; apex blunt with 1 robust seta subapically and 2 robust setae (1 large and 1 small) apically.

***Uropod 3*** (Fig. 5G): shortest; peduncle 0.8 times as long as that of uropod 2, with 1 medial seta dorsally; rami lancerolate; inner ramus 1.6 times as long as peduncle, with 7 robust and 8 plumose setae medially and 7 robust setae laterally; outer ramus slightly shorter than inner ramus, with 6 robust and 5 plumose setae medially and 5 robust setae laterally.

***Telson*** (Fig. 5H): shallowly cleft (about 1/4 length), with 1 pair of robust setae proximally, with single or paired simple setae on surface; each apex with 1 notch and 1 simple seta subapically.

***Oostegites*** (Fig. 6A–D): present on gnathopod 2 to pereopod 5, with numerous long setae marginally; that of gnathopod 2 largest; that of pereopod 3 larger than that of pereopod 4, both dilated distally; that of pereopod 5 sublinear, smallest.

###### Male.

Unknown.

###### Remarks.

*Eusiroides pilopalpus* sp. n. is very similar to *Eusiroides japonica* Hirayama, 1985. It can be readily distinguished from *Eusiroides japonica* by the following characteristic features: (1) degree of setation of peduncular articles 4 and 5 on antenna 2 is weaker than that of *Eusiroides japonica*; (2) anterior and posterior margins of peduncular article 5 on antenna 2 are parallel in *Eusiroides pilopalpus* sp. n. (vs. margins are gradually diminish distally, and anterodistal and posterodistal corners are produced in *Eusiroides japonica*); (3) mandibular palp article 3 has a group of brush-like setae on the lateral surface (vs. several rows of minute setae in *Eusiroides japonica*); (4) expanded medial lobe of mandibular palp article 2 is not swollen in *Eusiroides pilopalpus* sp. n. (vs. swollen medially in *Eusiroides japonica*); (5) inner plate on maxilla 2 is not enlarged in *Eusiroides pilopalpus* sp. n. (vs. distinctly enlarged in *Eusiroides japonica*); (6) maxillipedal palp article 3 is covered with fine setae on its distal surface (vs. transverse rows in *Eusiroides japonica*); (7) anterior lobe of ischium on gnathopods 1 and 2 is well-developed in *Eusiroides pilopalpus* sp. n. (vs. moderately developed in *Eusiroides japonica*), (8) acute protrusion at the posterodistal corner of merus on gnathopods 1 and 2 is larger than that of *Eusiroides japonica*; (9) length of the dactylus on gnathopods 1 and 2 is longer than that of *Eusiroides japonica*; (10) posterior lobe of ischium on pereopods 5–7 is acutely produced distally; (11) merus, carpus, and propodus on pereopods 5–7 are more slender than those of *Eusiroides japonica*; (12) both pleonal epimera 1 and 2 have oblique ridges on their lateral surfaces (vs. the ridges are not present in *Eusiroides japonica*) and posterior margin of pleonal epimeron 3 has 11 serrations (vs. seven in *Eusiroides japonica*); (13) the outer ramus on uropod 2 has three dorsal setae medially (vs. dorsal setae are absent in *Eusiroides japonica*); and (14) the distal fourth of the telson is cleft in *Eusiroides pilopalpus* sp. n., (vs. cleft beyond the distal half in *Eusiroides japonica*) (Hirayama 1985).

*Eusiroides pilopalpus* sp. n. shares several characters with *Eusiroides diplonyx* Walker, 1909. However, *Eusiroides diplonyx* can be easily discriminated from its congeners because it has stout and round-ended locking setae on pereopods 3 and 4 (Walker 1909; Pirlot 1936; Barnard JL 1970; Myers 1985). These are not observed at *Eusiroides pilopalpus* sp. n. in this study. Furthermore, *Eusiroides pilopalpus* sp. n. is different from *Eusiroides diplonyx* in the following ways: (1) inner plate on maxilla 2 is not enlarged in *Eusiroides pilopalpus* sp. n. (vs. the inner plate of maxilla 2 is larger than the outer plate in *Eusiroides diplonyx*); (2) one group of fine setae on the surface of mandibular palp article 3 is present in *Eusiroides pilopalpus* sp. n. (vs. absent in *Eusiroides diplonyx*); (3) posterior lobe of the ischium on pereopods 5–7 of *Eusiroides pilopalpus* is more acute than that of *Eusiroides diplonyx*; and (4) the telson of *Eusiroides pilopalpus* sp. n. is cleft to one fourth of the length (vs. more than half the length in *Eusiroides diplonyx*) (Walker 1909; Pirlot 1936; Barnard JL 1970; Myers 1985; Ren 2012).

#### Genus *Paramoera* Miers, 1875

##### Paramoera dentipleurae sp. n.

Taxon classificationAnimaliaAmphipodaPontogeneiidae

http://zoobank.org/D0556EA9-F25D-458F-9FDD-EEAFACAEA53F

**Figure 7.**
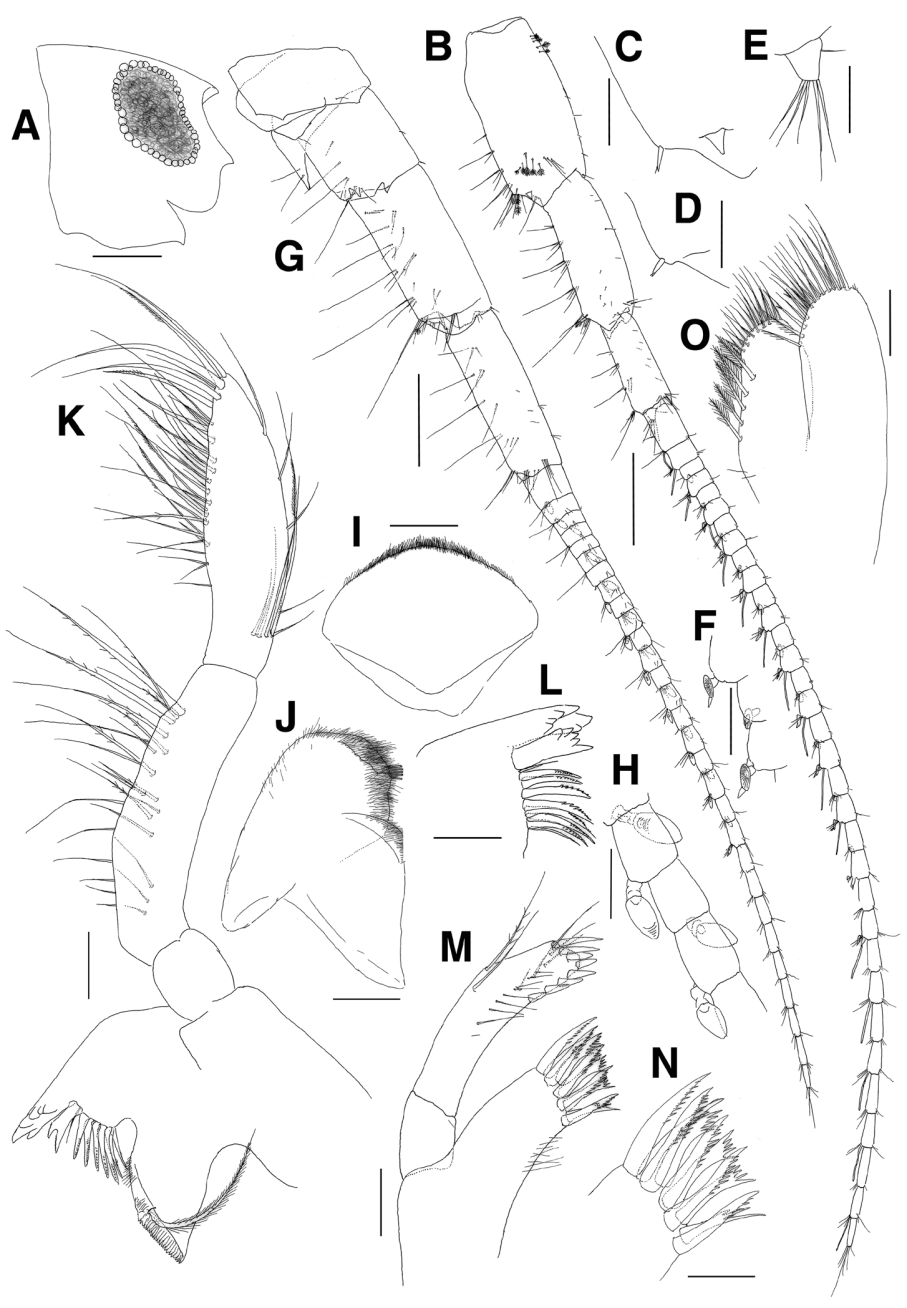
*Paramoera dentipleurae* sp. n., holotype. **A** Head **B** Antenna 1 **C** Posterodistal part of peduncular article 1 on antenna 1 **D** Posterodistal part of peduncular article 2 on antenna 1 **E** Accessory flagellum **F** Calceoli of flagellum on antenna 1 **G** Antenna 2 **H** Calceoli of flagellum on antenna 2 **I** Upper lip **J** Lower lip **K** Right mandible **L** Left mandible **M** Maxilla 1 **N** Dentate apical setae of outer plate on maxilla 1 **O** Maxilla 2. Scale bars 0.05 mm (**C–F, H–M, O**), 0.1 mm (**N**), 0.2 mm (**A, B, G**).

**Figure 8.**
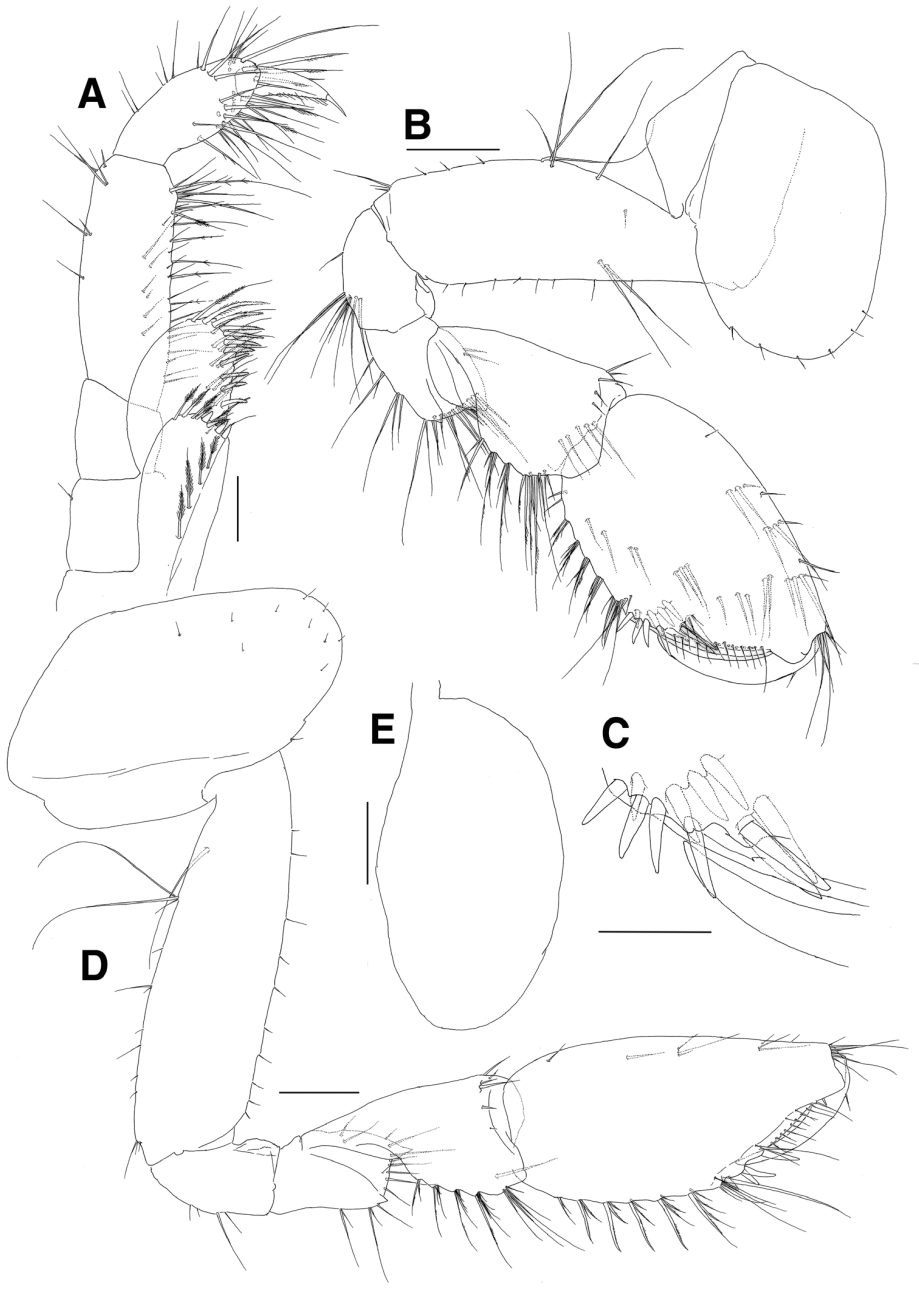
*Paramoera dentipleurae* sp. n., holotype. **A** Maxilliped **B** Gnathopod 1 **C** Setae of posterodistal margin of propodus on gnathopod 1 **D** Gnathopod 2 **E** Coxal gill of gnathopod 2. Scale bars 0.05 mm (**A, C**), 0.1 mm (**B, D, E**).

**Figure 9.**
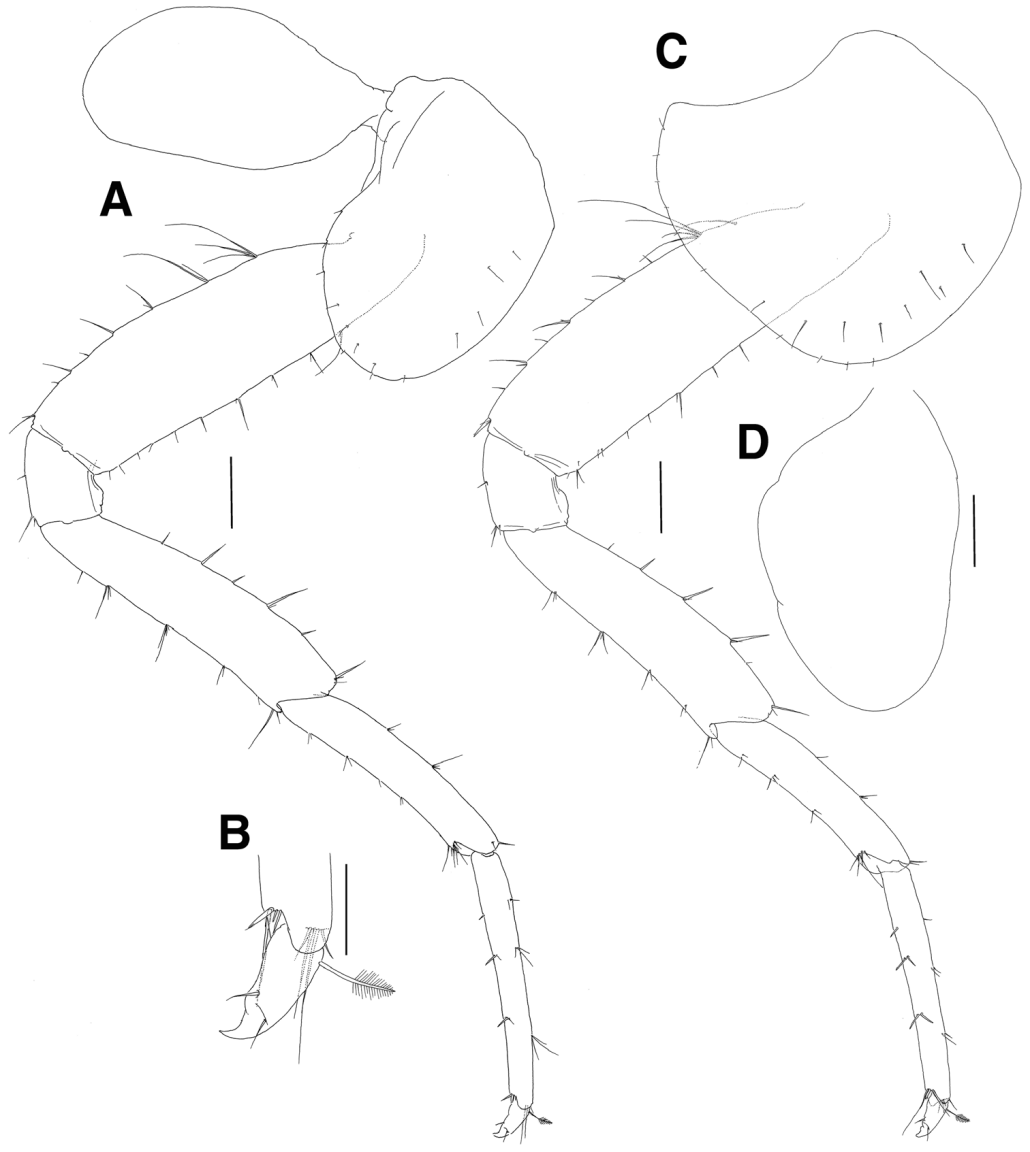
*Paramoera dentipleurae* sp. n., holotype. **A** Pereopod 3 **B** Dactylus of pereopod 3 **C** Pereopod 4 **D** Coxal gill of pereopod 4. Scale bars 0.05 mm (**B**), 0.2 mm (**A, C, D**).

**Figure 10.**
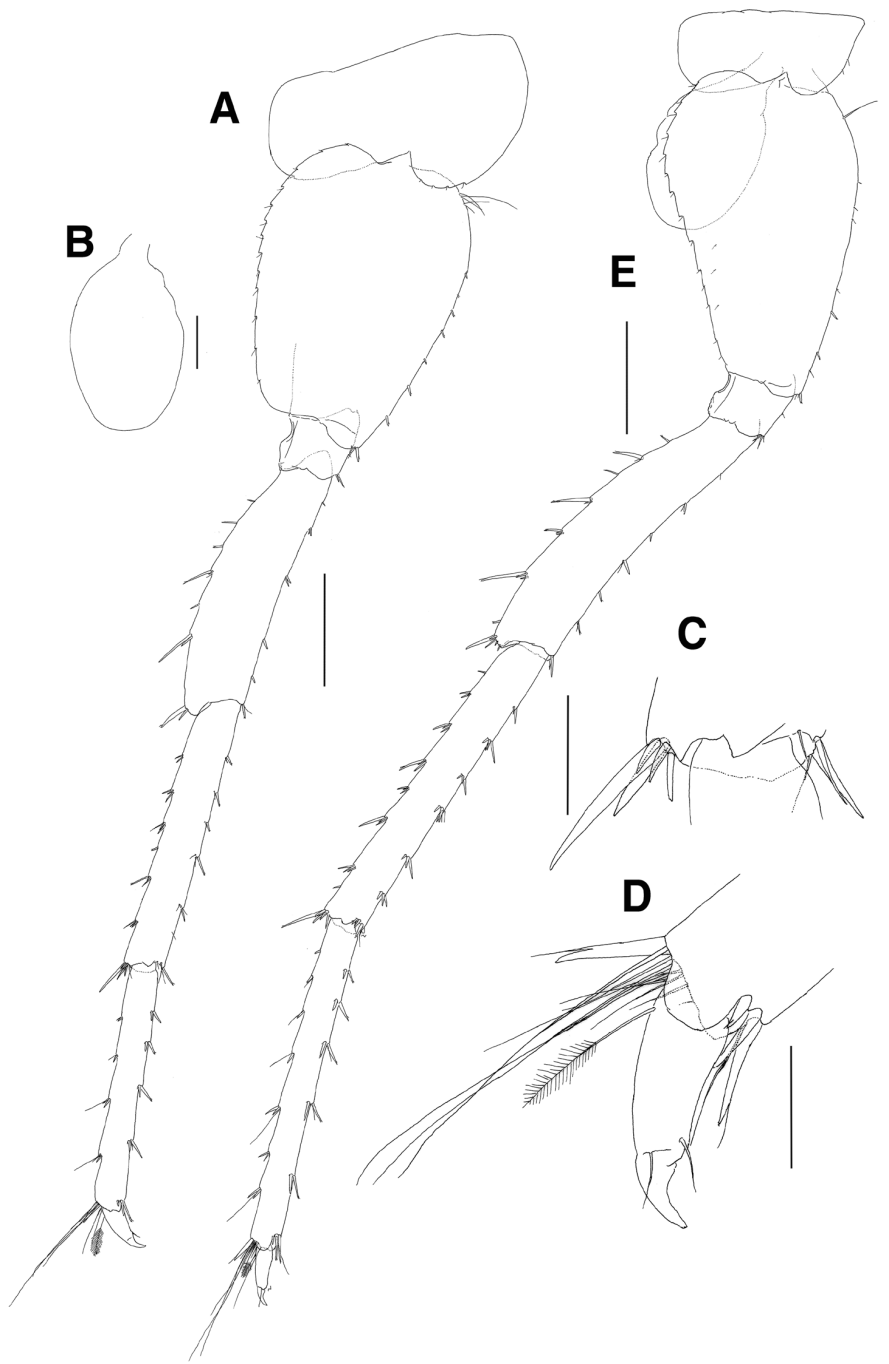
*Paramoera dentipleurae* sp. n., holotype. **A** Pereopod 5 **B** Coxal gill on pereopod 5 **C** Distal part of carpus on pereopod 5 **D** Dactylus on pereopod 5 **E** Pereopod 6. Scale bars 0.1 mm (**B**), 0.05 mm (**C, D**), 0.2 mm (**A, E**).

**Figure 11.**
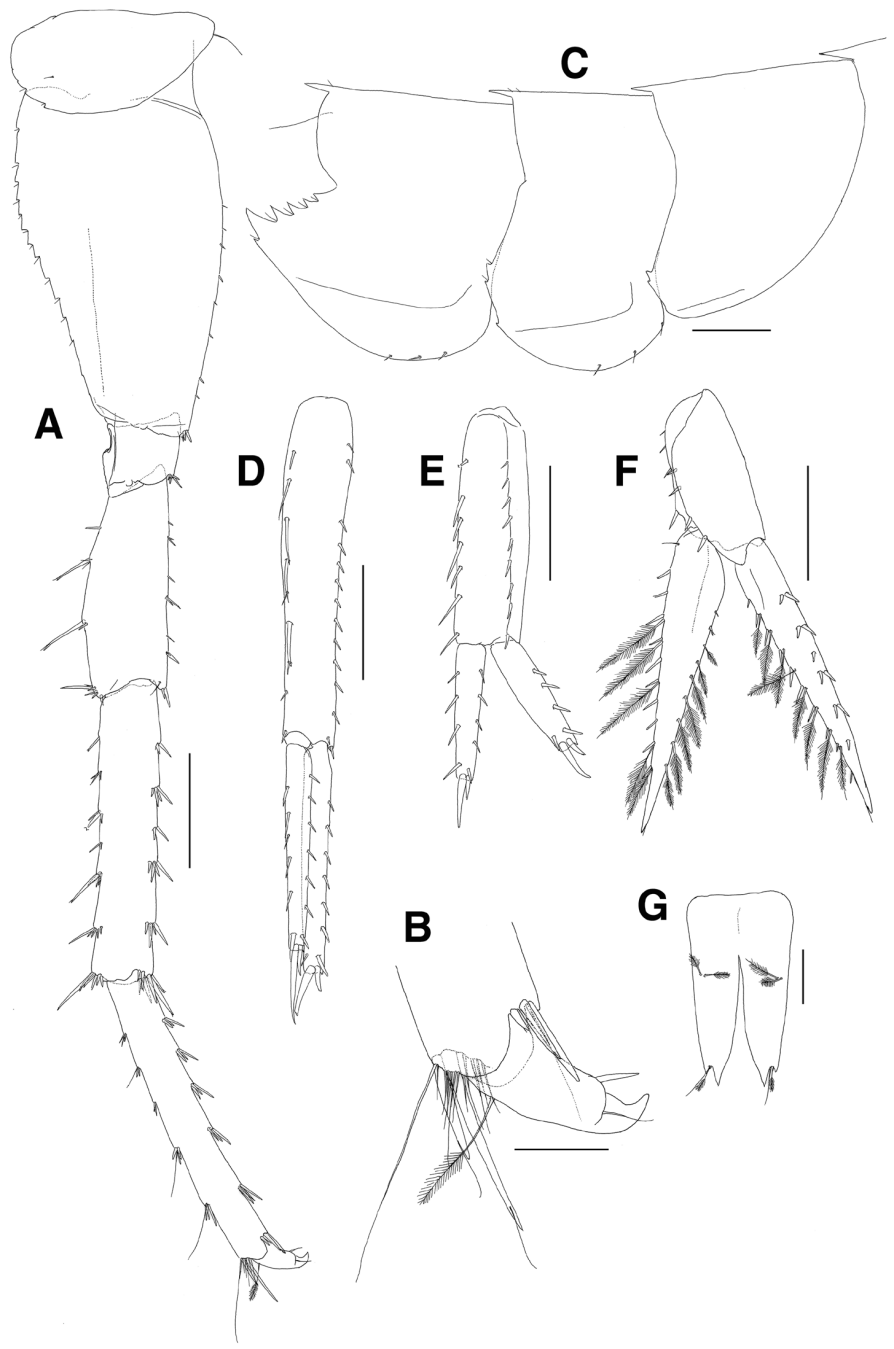
*Paramoera dentipleurae* sp. n., holotype. **A** Pereopod 7 **B** Dactylus on pereopod 7 **C** Pleonal epimera **D** Uropod 1 **E** Uropod 2 **F** Uropod 3 **G** Telson. Scale bars 0.05 mm (**B**), 0.1 mm (**G**), 0.2 mm (**A, C–F**).

Figs 7 , 8 , 9 , 10 , 11

###### Type locality.

Jeju Island, South Korea, 33°14'23"N 126°34'59"E, sublittoral (average depth 24 m).

###### Material examined.

Holotype: NIBRIV0000328602, adult female, 7.6 mm, collected from the type locality on 30 Nov 2012 by Prof. H.-Y. Soh.

###### Etymology.

The composite epithet of the species name of *dentipleurae* is a combination of Latin *dens*, Gen. *dentis* (meaning ‘teeth’ or ‘serration’) and *pleurae* (indicating ‘pleonal epimera’). Noun in apposition.

###### Diagnosis.

Head with short rostrum; lateral cephalic lobes not mammilliform, with sinusoid upper part; inferior antennal sinus forming deep notch, lower margin produced forward; eyes large, reniform. Antenna 1 with stout peduncular articles; accessory flagellum uni-articulate, short, scale-like. Antenna 2 slightly shorter than antenna 1. Lower lip outer lobe with 5 bifid setae mediodistally. Right mandible with 6-dentate incisor and bifid lacinia mobilis. Left mandible with 6-dentate incisor and 5-dentate lacinia mobilis. Maxilla 1 outer plate with 10 dentate robust setae apically; palp bi-articulate, apex beyond apical setae of outer plate. Maxilla 2 inner plate shorter than outer plate. Maxilliped outer plate as long as inner plate, with long serrate setae along apex and medial margins submarginally; palp articles 2 and 3 slender. Gnathopods 1 and 2 moderately subchelate. Gnathopod 1 palm oblique, with robust defining setae of 1, 1, 3 laterally and 2, 3, 1 medially in formulae. Gnathopod 2 palm more oblique than in gnathopod 1, with robust defining setae laterally in formula 1, 2, 1. Pereopods 3–7 dactylus short, with blunt protrusion bearing 1 seta on inner margin, apex curved and claw-like. Pereopods 5–7 elongate and slender. Pereopod 7 basis expanded posteriorly, angulate proximally and diminished distally. Pereonite 7 and pleonites 1–3 carinate dorsally. Pleonal epimeron 1 with 2 naked serrations and 1 weak notch posteriorly; epimeron 2 with 2 serrations bearing 1 minute seta and 1 naked notch posteriorly; epimeron 3 largest, weakly upturned posterodistally, with 5 serrations and 1 distal small notch bearing 1 minute seta posteriorly. Uropod 1 slender; peduncle longer than rami. Uropod 2 shortest. Uropod 3 rami lanceolate, longer than peduncle. Telson deeply cleft (approximately 3/4 length).

###### Description of holotype male.

***Head*** (Fig. 7A): rostrum short; lateral cephalic lobes not mammilliform, with sinusoid upper part; inferior antennal sinus forming deep notch, lower margin produced forward; eyes large, reniform.

***Antenna 1*** (Fig. 7B–F): peduncular articles stout, with length ratio of 1.0:0.8:0.5 in peduncular articles 1–3, each article with 4–6 groups of elongate setae along posterior margin; peduncular article 1 with 1 robust seta posterodistally and with small triangular lobe on surface posterodistally; peduncular article 2 also with 1 robust seta posterodistally; peduncular article 3 without posterodistal setae; accessory flagellum uni-articulate, short, scale-like, with 5 simple setae on apex and 1 simple seta anteroproximally; flagellum 33-articulate, 2.0 times as long as peduncular articles 1–3 combined, proximal article longest, with aesthetasc present on every alternate article and calceoli present from 3^rd^ to 24^th^ articles posterodistally.

***Antenna 2*** (Fig. 7G, H): slightly shorter than antenna 1; gland cone produced with 2 simple setae; peduncular articles stout, with length ratio of 1.0:1.7:1.8 in peduncular articles 3–5; flagellum 29-articulate, 1.6 times as long as peduncular articles 3–5 combined, proximal article longest, with calceoli present from 1^st^ to 20^th^ articles.

***Upper lip*** (Fig. 7I): triangular, apical margin convex, covered with fine setae distally.

***Lower lip*** (Fig. 7J): with well-developed mandibular processes bearing round apices; outer lobe apically round with 5 bifid setae mediodistally; inner lobe weak.

Right mandible (Fig. 7K): with 6-dentate incisor and bifid lacinia mobilis; accessory setal row lined with 1 simple, 5 serrate and 2 simple setae in turn; molar triturative, columnar, with 1 long plumose seta; palp enlarged, 3-articulate; palp article 1 shortest; palp article 2 lined with plumose setae on surface and medial margin; palp article 3 brush-like, with many simple, plumose and serrate setae along apex and medial margin.

***Left mandible*** (Fig. 7L): with 6-dentate incisor and 5-dentate lacinia mobilis; accessory setal row with 1 simple, 7 serrate setae and 1 simple seta in turn.

***Maxilla 1*** (Fig. 7M, N): outer plate with 10 dentate setae apically; palp long, beyond apical setae of outer plate, article 2 with 1 row of 9 setae on surface and 8 robust setae along apical and mediodistal margins, with 1 plumose and 1 simple seta at centre of lateral margin.

***Maxilla 2*** (Fig. 7O): inner plate with 1 oblique row of 5 plumose setae on medial margin, with apical and subapical setae; outer plate with 2 serrate and 2 forked setae on mediodistal margin, with apical and subapical setae.

***Maxilliped*** (Fig. 8A): inner plate subrectangular, swollen laterodistally, with 3 robust setae apically, with plumose setae on apex and mediodistal margin; outer plate as long as inner plate, convex laterally and wider than inner plate, with elongate plumose setae along apex and medial margin submarginally; palp elongate, slender, 4-articulate; article 2 subrectangular, longest, lined with long forked and simple setae on medial margin; article 3 thumb-shaped, with forked, serrate and simple setae on distal surface and margin; article 4 falcate, 0.8 times as long as article 3, lined with setae on medial margin, with 1 elongate seta apically.

***Gnathopod 1*** (Fig. 8B, C): subchelate; coxa subrectangular, ventral margin round with 5 minute setae and with 1 small notch bearing 1 minute seta posteroventrally; basis anterior margin straight with 8 minute setae, with 1 pair of elongate setae on medial surface anteroproximally, posterior margin with 1 elongate seta proximally, lined with 1 group of 4 elongate setae and with 3 minute setae, with 1 group of 3 simple setae subdistally; ischium with moderate anterior lobe, posterior margin convex with 1 simple seta and 1 group of 9 elongate setae; merus rectangular, as long as ischium, forming groove anterodistally, with medial and lateral protrusions distally, with 1 pair of short setae medially, with elongate setae in formula 1, 2, 3, 2 on posterior margin, lined with 14 long setae on distal margin; carpus longer than merus, with 1 lateral row of 5 setae anterodistally and 1 medial row of 5 setae on distal margin, posterior margin broadly lobed and weakly crenulate, with 4 groups of serrate and simple setae; propodus ovoid, as long as basis, with 4 setae on anterior margin, with 1 group of 6 simple setae at anterodistal corner, with 5 groups of serrate setae on weakly crenulate posterior margin, with setae of various combinations on medial surface, palm oblique with medial and lateral rows of simple setae, with robust defining setae of in formulae 1, 1, 3 laterally and 2, 3, 1 medially; dactylus falcate, stout, slightly shorter than palm.

***Gnathopod 2*** (Fig. 8D, E): also moderately subchelate, similar to gnathopod 1 in size; coxa subrectangular, with 3 minute setae on round ventral margin anteriorly, with 2 small notches posteroventrally, with 7 minute setae on anterodistal surface, coxal gill ovoid and large, slightly shorter than basis; basis anterior margin slightly convex lined with 10 minute setae, posterior margin with 1 elongate setae proximally and 1 group of 3 simple setae (2 elongate and 1 short) at proximal 1/3, lined with 7 simple setae and 1 group of 3 simple setae subdistally at distal half; ischium with moderate anterior lobe, posterior margin convex with 4 setae; merus as long as ischium, forming groove anterodistally, with medial and lateral protrusions distally, with 2 pairs of simple setae on anterior margin medially, with 1 seta and 2 pairs of setae on posterior margin distally, with 6 elongate setae on distal margin; carpus longer than merus, with 1 lateral row of 6 setae anterodistally, posterior margin broadly lobed and weakly crenulate with 5 groups of serrate and simple setae; propodus ovoid, as long as basis, with 4 simple setae on anterior margin, with 1 group of 6 simple setae at anterodistal corner, weakly crenulate posteriorly with 7 groups of serrate setae, palm more oblique than in gnathopod 1, with medial and lateral rows of simple setae, with robust defining setae laterally in formula 1, 2, 1; dactylus falcate, stout, slightly shorter than palm.

***Pereopod 3*** (Fig. 9A, B): coxa subrectangular, convex anteriorly, with 4 minute setae on anterior surface, ventral margin round with 3 marginal and 5 submarginal setae, with 2 small notches bearing 1 minute seta at posteroventral corner, posterior margin short, coxal gill ovoid and large, as long as coxa; basis anterior margin straight with 4 pairs and 6 minute setae, posterior margin setose; ischium short, with moderate anterior lobe, with 1 minute seta on posterior margin and 1 pair of simple setae at posterodistal corner; merus 0.7 times as long as basis, anterior margin weakly spinose and slightly expanded distally, with 1 group of 3 simple setae at anterodistal corner, posterior margin weakly setose; carpus not expanded and slightly curved, with 2 groups of simple setae on anterior margin and 1 pair of simple setae at anterodistal corner, with 1 minute and 3 pairs of simple setae on posterior margin, with several minute setae at posterodistal corner, distal margin round; propodus slender, 1.1 times as long as carpus, with 3 groups of simple setae on anterior margin and 1 row of 4 setae at anterodistal corner, with 1 small seta and 2 pair of setae on posterior margin, with 1 elongate, 1 robust and 2 moderate setae at posterodistal corner; dactylus short, with blunt protrusion bearing 1 seta on inner margin, apex curved and claw-like.

***Pereopod 4*** (Fig. 9C, D): coxa subquadrate, with acutely produced posterior margin; dactylus with blunt protrusion on inner margin but weaker than that of pereopod 3; other articles similar to those of pereopod 3 except for additional 1 robust seta at posterodistal corner of basis.

***Pereopod 5*** (Fig. 10A–D): elongate and slender; coxa bilobed, anterior lobe expanded downwards with 4 minute setae on ventral margin, posterior lobe not expanded with 1 small notch bearing 1 minute seta at posteroventral corner, coxal gill ovoid and small; basis subovoid, anterior margin convex with 1 group of 5 elongate setae proximally and lined with 7 robust setae, anterodistal corner weakly lobed downwards with 1 pair of robust setae, posterior margin moderately expanded with 13 weak serrations bearing 1 minute seta; ischium short, anterodistal corner weakly lobed downwards with 1 pair of setae, posterior lobe moderate; merus as long as basis, with short robust setae on anterior margin and with 1 pair of robust and simple setae at anterodistal corner, posterior margin weakly expanded and setose with robust setae, posterodistal corner slightly produced with 1 pair of unequal setae; carpus linear and slender, as long as merus, margins weakly setose, anterodistal and posterodistal corners with robust and simple setae; propodus also linear, 0.9 times as long as carpus, with 4 pairs of robust setae on anterior margin and with 1 group of 4 setae (1 simple elongate and 3 robust) at anterodistal corner, with 4 groups of robust and simple setae on posterior margin and with 10 setae (1 robust and 9 simple) at posterodistal corner; dactylus short, with blunt protrusion bearing 1 seta on inner margin, with curved and claw-like apex.

***Pereopod 6*** (Fig. 10E): elongate and slender, longer than pereopod 5; coxa bilobed, smaller than that of pereopod 5, anterior lobe expanded downwards with 2 minute setae anteriorly, posterior lobe not expanded with notch bearing 1 minute seta at posteroventral corner, coxal gill ovoid and slightly smaller than that of pereopod 5; basis as long as that of pereopod 5, anterior margin convex with 1 elongate seta proximally and irregular spaced 7 robust and simple setae, anterodistal corner weakly lobed downwards with 1 robust seta, posterior margin expanded proximally and diminished distally, with 13 weak serrations bearing 1 minute seta and 2 minute setae distally; ischium short, anterodistal corner weakly lobed downwards with 1 group of 3 setae, posterior lobe moderate; merus as long as basis, setose with robust and simple setae on anterior margin, with 1 pair of setae at anterodistal corner, posterior margin weakly expanded and setose with elongate and short robust setae irregularly, posterodistal corner slightly produced with 1 group of 5 setae; carpus linear and slender, 1.1 times as long as merus, margins weakly setose, anterodistal and posterodistal corners with robust and simple setae; propodus also linear, as long as carpus, with 5 groups of robust and simple setae on anterior margin, and with 1 group of 4 setae (1 simple elongate and 3 robust) at anterodistal corner, posterior margin armed with setae of various combinations, with 9 setae (8 simple elongate and 1 robust) at posterodistal corner; dactylus short, with blunt protrusion bearing 1 seta on inner margin, with curved and claw-like apex.

***Pereopod 7*** (Fig. 11A, B): shorter but stouter than pereopod 6; coxa unilobed, with 1 simple seta at anterior corner, with 2 notches bearing 1 minute seta and 2 small naked notches on posterior margin; basis larger than that of pereopod 6, anterior margin widened with irregular spaced 8 minute setae, anterodistal corner a little lobed distally with 1 group of 3 robust setae, posterior margin expanded, angulate proximally and diminished distally, with 12 weak serrations bearing 1 minute seta and 2 naked serrations distally; ischium short, anterodistal corner weakly lobed downwards with 1 group of 5 robust setae, posterior lobe moderate; merus short, 0.7 times as long as basis, anterior margin straight and setose with single or paired setae, anterodistal corner weakly produced with 1 subapical simple seta and 1 pair of robust apical setae, posterior margin slightly expanded and setose with irregular elongate and short robust setae, posterodistal corner slightly produced with 1 group of 6 robust setae (1 elongate and 5 moderate); carpus linear, as long as basis, with 5 groups of robust setae on anterior margin and with 1 group of 7 robust setae at anterodistal corner, with 1 single and 5 groups of robust setae on posterior margin and with 1 group of 6 robust setae at posterodistal corner; propodus also linear, as long as carpus, with 5 groups of 3 robust setae on anterior margin and 1 group of 4 setae (1 simple elongate and 3 robust) at anterodistal corner, posterior margin armed with setae of various combinations, with 13 setae (2 robust and 11 simple) at posterodistal corner; dactylus short, with blunt protrusion bearing 1 seta on inner margin, with curved and claw-like apex.

***Pereonite 7 and pleonites 1–3*** (Fig. 11C): each carinate dorsally.

***Pleonal epimera*** (Fig. 11C): epimeron 1 with 2 naked serrations and 1 weak notch on posterior margin; epimeron 2 round ventrally, with 3 setae on anterior half of ventral margin, with 2 serrations bearing 1 minute seta and 1 naked notch on posterior margin; epimeron 3 largest, weakly upturned posterodistally, with 3 setae on ventral margin, with 5 serrations and 1 distal small notch bearing 1 minute seta on posterior margin.

***Uropod 1*** (Fig. 11D): slender; peduncle longer than rami, with 14 lateral and 9 medial robust setae dorsally; outer ramus with 6 lateral robust setae dorsally, apex blunt with 2 subapical and 2 apical robust setae; inner ramus shorter than outer ramus, with 6 lateral and 5 medial robust setae dorsally, apex blunt with 2 subapical and 2 apical robust setae.

***Uropod 2*** (Fig. 11E): shortest; peduncle 0.7 times as long as that of uropod 1, with 9 lateral and 7 medial robust setae dorsally; outer ramus shorter than peduncle, with 4 lateral robust setae dorsally, apex blunt with 2 subapical and 2 apical robust setae; inner ramus subequal to outer ramus, with 4 lateral and 3 medial robust setae dorsally, apex blunt with 2 subapical and 2 apical robust setae.

***Uropod 3*** (Fig. 11F): peduncle 0.7 times as long as that of uropod 2, with 4 medial and 4 lateral robust setae dorsally; rami lanceolate; outer ramus 2.0 times as long as peduncle, with 4 pairs and 2 single robust setae along lateral margin, with 2 single robust setae, 7 pairs of robust and plumose setae, and 1 minute subapical seta along medial margin; inner ramus slightly longer than outer ramus, with 2 proximal single setae, 7 plumose setae and 1 minute subapical seta on lateral margin, with 1 proximal simple seta, 10 robust and 5 plumose setae on medial margin.

***Telson*** (Fig. 11G): deeply cleft (about 3/4 length), each lobe with notch bearing 1 pair of 1 plumose and 1 long simple seta on apex, with 1 pair of plumose setae on dorsal surface.

###### Female.

Unknown.

###### Remarks.

*Paramoera dentipleurae* sp. n. is very similar to *Paramoera tridentata* Bulycheva, 1952 and *Paramoera hanamurai* Hirayama, 1990 in the following characteristics: (1) the anterior cephalic lobe is sinusoid; (2) pereopods 5–7 are more slender than those of other congeners; (3) the posterior lobe of basis on pereopods 6–7 is largely expanded proximally and diminished distally; (4) epimera 1–3 are carinate dorsally; and (5) epimeron 3 is prominently expanded backward with very similar serration pattern on the posterior margin (Bulycheva 1952; Hirayama 1990). However, *Paramoera dentipleurae* sp. n. can be readily distinguished from *Paramoera tridentata* by the following features: (1) the inferior antennal sinus is deeply cleft and its lower part is produced forward in *Paramoera dentipleurae* sp. n., whereas it is concaved quadrately in *Paramoera tridentata*; (2) the formulae of robust defining setae at the palmar corners on gnathopods 1 and 2 are complex in *Paramoera dentipleurae* sp. n. (1, 1, 3 for lateral and 2, 3, 1 for medial setae on gnathopod 1 and 1, 2, 1 for lateral setae on gnathopod 2), whereas *Paramoera tridentata* has only two lateral setae on gnathopods 1 and 2, respectively; (3) the serrations of each posterior margin of the basis on pereopods 5–7 are weaker in *Paramoera tridentata*; and (4) the dorsal carina on pereon 7 is absent in *Paramoera tridentata* (Bulycheva 1952; Labay 2012).

*Paramoera dentipleurae* sp. n. can be discriminated from *Paramoera hanamurai* by the following features: (1) upper and lower parts of the inferior antennal sinus do not overlap in *Paramoera dentipleurae* sp. n.; (2) antenna 1 is longer than antenna 2 in *Paramoera dentipleurae* sp. n., but antenna 2 is longer than antenna 1 in *Paramoera hanamurai*; (3) setations of the peduncular articles on antennae 1 and 2 of *Paramoera dentipleurae* sp. n. are weaker; (4) accessory flagellum has five apical setae in *Paramoera dentipleurae* sp. n., but it has only two apical setae in *Paramoera hanamurai*; (5) formulae of the robust defining setae at the palmar corners on gnathopods 1 and 2 are different from each other (1, 1, 3 for lateral setae and 2, 3, 1 for medial setae on gnathopod 1, and 1, 2, 1 for lateral setae in gnathopod 2 in *Paramoera dentipleurae* sp. n., whereas there are three rows of three setae on the medial surface of gnathopod 1 and without setae on gnathopod 2 in *Paramoera hanamurai*); (6) posterior margin of basis on pereopod 5 is more expanded distally and the length of the merus is shorter in *Paramoera hanamurai*; (7) serration patterns are weaker and the posterodistal lobes are indistinct for basis on pereopods 5–7 in *Paramoera dentipleurae* sp. n. compared to *Paramoera hanamurai*; (8) each dorsal margin from pereon 7 to pleon 3 has a distal carina in *Paramoera dentipleurae* sp. n., whereas the distal carina is absent in *Paramoera hanamurai*; and (9) serration patterns of the posterior margins on the pleonal epimera are simpler in *Paramoera hanamurai* compared to those in *Paramoera dentipleurae* sp. n. (Hirayama 1990).

## Supplementary Material

XML Treatment for Eusiroides pilopalpus

XML Treatment for Paramoera dentipleurae
